# The Voice

**Published:** 1871-01

**Authors:** 


### THE VOICE.

Sound is a series of air-waves, extending and widening as in the water when a pebble is thrown into it. Musical sounds travel farther than others by one half. A strong, healthy man can "fill" a room measuring seventy-five feet each way; if he sings, he can fill a room half as large again.

There may be a hum of voices in a room, and a fife playing at the same time; you may not be able to hear the fife in the room, the hum drowns it; this hum will travel a hundred yards perhaps; but far beyond that you can hear the fife with great distinctness. At the same time, as between speaking in a monotonous or sing-song tone, and a distinct enunciation with an appreciable interval between each word and syllable, there can be no comparison as to the greater ease in understanding the latter, although the volume of sound in the former is much greater. Many a public speaker is pleasurably understood with much less power expended in vocalization, while another, with stentorian lungs, fails to convey his ideas to the hearer, the difference being in the manner of enunciation.

Monotonous speakers are heard painfully, because the sounding of one word runs into another, and the attention is kept on the stretch all the time to catch the intervals, which it is necessary to do in order to comprehend the idea. A certain method of avoiding this great and unfortunate mistake is for the speaker himself so to pronounce each word that his own ear can easily measure a distinct interval, not only between each word, but between every syllable. If a public speaker will pay the proper attention, he will discover that there exists a certain instinct within him, which will enable him to feel whether his voice fills the room and reaches the whole audience.

A blind man was once sent to an office to deliver a message. On returning, he reported that on opening the door he found no person there. On being asked how he knew it, he replied that the rebound of the voice, its force and character, were different in an empty room and one more or less full. There is a ring in an empty room; if full, the voice falls more like a thug or muffled sound.

A man may cast a pebble fifty yards, and fifty other men of equal strength and skill could each cast it fifty yards but no one of the fifty can throw that pebble farther than fifty yards. So five hundred voices, although very loud within the sphere of one voice, will not be heard farther than one voice of the five hundred. Thus a man may be singing in a crowd where the general hum prevents him hearing himself, yet he will be heard at a distance far beyond that which the hum reaches, because a musical note travels farther than one of prose.

To speak without an effort of mind or voice or person, the whole consciousness must be absorbed in the subject itself. Then only can a man speak with nature's eloquence. He must for the instant feel that his subject is overwhelmingly real, overwhelmingly important; hence it is that all the "arts" of oratory, such as studied gestures, attitudes, attention to the modes of breathing, and efforts to speak so as not to exhaust the lungs fully, are so much wastes of physical power, as well as moral force, because just that much attention is taken from the subject, and deprives it of its life. The oratory of the heart which is effective the world over, whether in assemblies civilized or savage, cultivated or all untutored, is that which comes from a man whose consciousness makes him feel that material and immortal destinies hang upon his utterances.

